# Spatiotemporal gait parameters in young individuals wearing an age simulation suit compared to healthy older individuals

**DOI:** 10.1186/s11556-022-00298-w

**Published:** 2022-11-18

**Authors:** Thea Laurentius, Johannes Quandel, Leo Cornelius Bollheimer, Steffen Leonhardt, Chuong Ngo, Markus Lüken

**Affiliations:** 1 Department of Geriatric Medicine, RWTH Aachen University Hospital, 52074 Aachen, Germany; 2 Medical Information Technology (MedIT), RWTH Aachen University, 52074 Aachen, Germany

**Keywords:** Age simulation suit, Artificially altered gait behavior, Spatiotemporal gait parameters, Gait in the elderly

## Abstract

**Introduction:**

Aging is accompanied by changes in muscle mass, strength and loss of sensory, visual and auditive functions. However, these changes do not occur linearly, most spatiotemporal gait parameters change with aging. Age simulation suits have been invented to give young people an impression of the implications of being older and may be a useful tool in the scientific setting for gerontology research to validate any study concept before it becomes a pilot study. The rationale behind this study was to investigate the effects of an age simulation suit on gait parameters in young healthy adults and to compare the altered gait with healthy older, community-dwelling citizens.

**Methods:**

Subjects were 14 healthy young adults (6 female) and 8 healthy older (4 female) individuals with a mean (± SD) age of 24.8 ± 3.4 years and 72 ± 1.9 years, respectively. After initial baseline measurements had been taken and a familiarization phase, the younger subjects walked for 15 min without and 15 min with an age simulation suit on an instrumented treadmill. The older subjects walked once for 15 min on the same treadmill without wearing an age simulation suit. The walking speed was self-selected for all subjects.

**Results:**

The age simulation suit reduced the walking speed from 4.1 ± 0.7 km/h to 3.3 ± 0.5 km/h (*p* < 0.001) in young adults with no differences compared to older adults (2.9 ± 0.6 km/h, *p* = 0.9). Step width increased from 8.7 ± 2.2 cm to 12.1 ± 2.2 cm (*p* < 0.001) and did not differ from older participants (11.1 ± 4.3 cm, *p* = 0.37). The stride length was reduced (132.6 ± 5.9 cm vs 118.1 +—6.6 cm, *p* < 0.001), but still did not match the old control group (94.5 ± 5.6 cm, *p* < 0.05). Wearing the suit increased thestride time of young subjects (from 1,152 to 1,316 ms, *p* < 0.001) and was different compared to the older control group (1,172 ms, *p* = 0.53). The coefficient of variation (COV) of spatiotemporal parameters did not differ between young (both not wearing the suit and wearing the suit) and older subjects. The standard deviation of lateral symmetry, an in-house marker from the instrumented treadmill that serves as a marker of gait variability, differed between young subjects without the suit and older subjects (5.89 ± 1.9 mm vs 14.6 ± 5.7 mm, *p* < 0.001) but not between young subjects wearing the suit and older subjects (16.4 ± 7.4 mm vs 14.6 ± 5.7 mm, *p* = 0.53).

**Conclusion:**

Wearing an age simulation suit while walking on a treadmill with a self-selected walking speed alters some, but not all, measured spatiotemporal parameters to approximate a gait pattern similar to that of an older individual.

## Highlights

Wearing an age simulation suit affects some, but not all gait parameters in young healthy adults.

## Introduction

According to the WHO report on aging and health, both the proportion and the absolute number of older people in populations around the world are increasing dramatically. Today, in Japan alone, the percentage of people 60 years or older exceeds 30%, whereas by 2050 many other countries will have equally high proportions [1]. The German Federal Office of Statistics [2] estimated that the percentage of people older than 60 years in Germany will rise from 27 (2014) to 38% (2050) and the percentage of people older than 80 years will rise from 6 to 13% within the same period. Aging is often accompanied by a loss of muscle mass, strength and function (sarcopenia) and a loss of sensory, visual or auditory function. These changes may occur in a non-linear manner, which results in very different levels of physical functioning in the older population [1]. Furthermore, most spatiotemporal gait parameters, such as preferred walking speed, cadence, step length, and step and stride time, deteriorate with aging, while gait variability measures seem to remain constant in healthy adults over time [3]. Age simulation suits were developed to demonstrate possible spatiotemporal and gait variability changes that may occur with aging. Nowadays, these suits are regularly used for the education of paramedics, nurses, physiotherapists and medical students and may be an inexpensive and useful tool to promote empathy towards the older population [4–6]. However, only few studies have defined the influence of the suit on younger subjects [7], e.g.).

Suits manufactured by multiple companies aim to simulate joint stiffness, visual or sensory impairments, increased kyphosis of the spine and other artificial impairments to mimic problems accompanied with aging [8]. The Age simulation suits could not only in the education of medical professionals but also as a reality check in many other parts of areas such as timing of pedestrian traffic lights, elevator doors or automatic doors in shopping malls.

Additionally, age simulation suits may also be a useful tool in the scientific setting for gerontology research to validate a study concept even before beginning a pilot study Lauenroth et al. [9] previously investigated the effect of one particular age simulation suit on a ground-level surface with a length of four meter and found that velocity and step time corresponded with gait characteristics of older subjects.

Regarding our study, it is hypothesized that wearing an age simulation suit changes the gait characteristics of young people. Furthermore, it is hypothesized that the altered gait can mimic the gait characteristics of older people. An additional interesting aspect of this study was to investigate whether the recalibration abilities of the younger participants to the movement limitations caused by the aging simulation suit would negate the intended effects of the suit in terms of gait parameter degradation. The adaption capabilities of the locomotor system are well described in the literature [10, 11], however, the effects of age simulation suits have not been investigated in this context yet.

## Methods

### Subjects

A total of 22 subjects (14 young and 8 older) were recruited via senior university lectures, advertisements in pharmacies, a database of participants of former studies and word of mouth. In order to include only young and old participants, younger participants were between 18 and 35 years old and older participants were at least 65 years old. Exclusion criteria were not being able to speak or understand German, known cognitive impairment, visual impairments (defined as degree of visual impairment that cannot be corrected with glasses to 80% of normal vision), sensory impairments, acute orthopaedic, cardiovascular or neurological diseases or acute pain. Exclusion criteria were assessed with questionnaires via self-disclosure of participants before other baseline characteristics were obtained. The study was approved by the blinded ethical committee information with all participants giving their written consent before participation.

### Age simulation suit

**Fig. 1:**
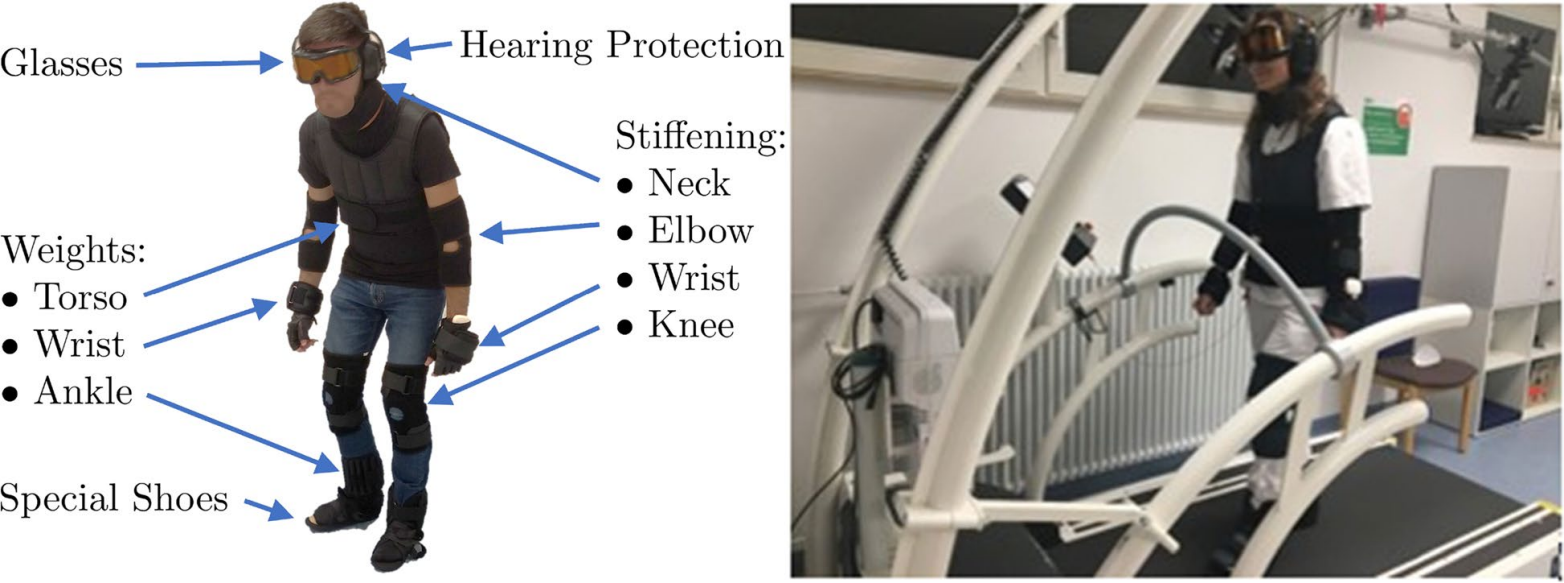
Schematic of the age simulation suit. Left: Young subject wearing the GERT explaining the single components of the aging simulation suit. Right: Young subject walking on the instrumented treadmill wearing the aging simulation suit. The slope of the platform was set at 0° for all trials and participants. Apart from common spatiotemporal parameters, the instrumented treadmill used in this experiment can obtain more possibly interesting data, such as lateral symmetry. Lateral symmetry describes the left/right shift of the center of pressure (COP) intersection point in chronological sequence in the cyclogram, taking all steps into account. A negative value indicates a shift to the left, and a positive value a shift to the right

In contrast to the older participants, younger participants wore an age simulation suit (GERT, Produkt + Projekt Wolfgang Moll, Niederstotzingen, Germany), which consists of a weighted vest (10.2 kg), weights for wrists (2 × 1.5 kg) and ankles (2 × 2.3 kg), a ruff, bandages around elbows and knees to impair joint mobility, glasses, ear protection and special shoes to simulate the loss of sensory function. The special shoes comprise a stiff shoe sole and a trapezoidal shape to inhibit the natural rolling movement of the foot. The total weight of the suit was approx. 19.8 kg. In summary, the suit aims to mimic physical and sensory difficulties experienced with aging (Fig. 1).

### Treadmill

To assess gait parameters, an instrumented treadmill (HP Cosmos, Germany) with a pressure platform (FDM-THQ Zebris Medical GmbH, Isny, Germany) was used (Fig. 1). Before starting the walking test, all participants were given five minutes of familiarization to get accustomed to walking on the treadmill as well as finding their desired walking speed. The walking speed was initialized with 3 km/h and gradually in- or decreased by the supervising investigator to approximate the individually preferred walking speed. After the trial had started the subjects were asked to walk at the determined treadmill velocity while being distracted as little as possible. After the familiarization phase, participants older than 65 were assigned to walk for 15 min with a self-selected pace on the instrumented treadmill, while the younger participants were asked to walk 15 min without and 15 min with an age simulation suit on the same treadmill. In between the trials, younger participants were given a break between their two walking trials to prevent fatigue.

### Data analysis

All resulting data are displayed with mean and standard deviation. In addition to spatiotemporal parameters, the coefficient of variation (COV) was calculated ((SD/mean)*100%) for some variables to observe the variability of certain gait parameters. Presenting the COV of lateral symmetry was not applicable due to very small positive and negative values andlarge standard deviations, presenting the COV of *lateral symmetry* was not applicable. Hence, the standard deviation of *lateral symmetry* was used for comparisons between groups. The Wilcoxon signed-rank test was used to compare baseline characteristics of all younger and older subjects and means of younger subjects without and with the age simulation suit in a pairwise manner. Wilcoxon rank-sum tests were applied to compare young subjects without and with the suit against the old participants. All statistical analyses and graphical representations were performed with R (R Studio, R version 3.6.2, www.r.project.org), with a significance level set at *p* < 0.05.

## Results

Results of baseline characteristics can be seen in Table 1. Except for body weight (*p* = 0.23) and days of longer walking per week (*p* = 0.37), younger and older participants differed in height, Body mass index, hours of sport per week and normal gait speed (obtained on the treadmill).

**Table 1:** Baseline characteristics (expressed as mean ± SD)

Baseline characteristics	Younger	Older	*p*-value
Sex	6 female, 8 male	4 female, 4 male	
Age	24.8 ± 3.4 yrs	72 ± 1.9 yrs	< 0.01
Weight	70.4 ± 9.8 kg	76.3 ± 10.7 kg	0.23
Height	180 ± 10 cm	170 ± 10 cm	< 0.01
Body mass index	21.9 ± 2.1	27.7 ± 3.1	< 0.01
Grip strength	44.2 ± 11.8 kg	30.9 ± 11 kg	0.01
hours sport/week	5.9 ± 3.8	2.7 ± 1.5	0.02
Days/week of walks > 1 h	4.4 ± 2.0	4.6 ± 1.8	0.37
Gait speed normal	4.1 ± 0.7 km/h	2.9 ± 0.6 km/h	< 0.01

**Fig. 2:**
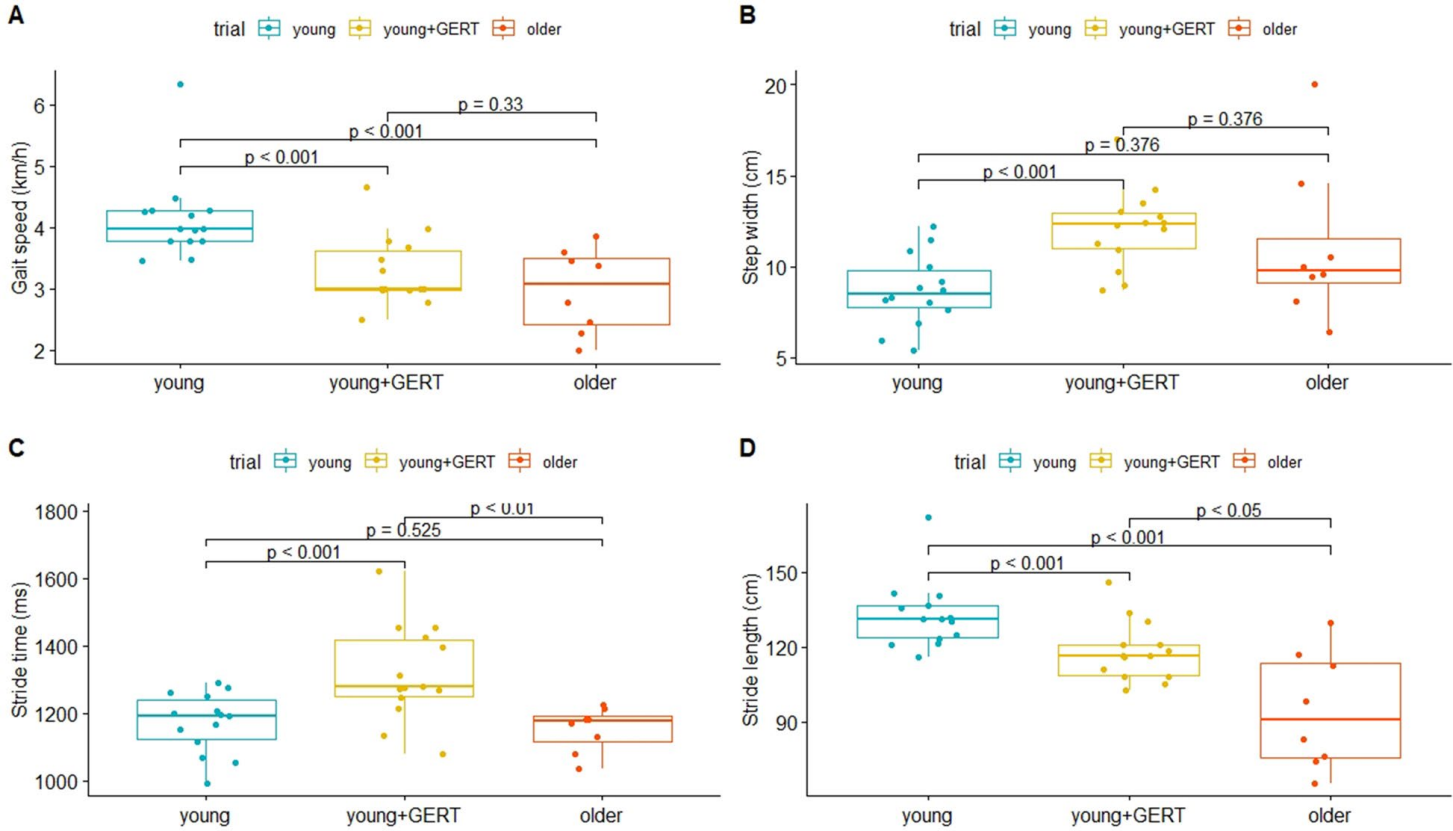
Gait parameters in young subjects with and without age simulation suit compared to older subjects. **A** Wearing the age simulation suit decreased the gait speed of younger subjects significantly (**B**) GERT increased the step width in younger participants. **C** Stride time was increased wearing GERT. **D** Stride length was reduced wearing GERT

Differences between younger people without wearing an age simulation suit (hereinafter referred to as “young”) or young people wearing an age simulation suit (hereinafter referred to as “young + GERT”) and older participants (hereinafter referred to as “older”) can be obtained in Table 2. Wearing the age simulation decreased the walking speed of younger participants from 4.1 ± 0.7 to 3.3 ± 0.5 km/h (*p* < 0.001) and differed not from walking speed obtained from older participants (2.9 ± 0.6 km/h, *p* = 0.33) (Fig. 2). Steps per minute (cadence) did not differ between normal walking of younger subjects without wearing GERT and older participants (*p* = 1), but wearing an age simulation suit significantly reduced the cadence between young participants walking normally (92 vs 103 steps/min, *p* < 0.001) and older participants (92 vs 105 steps/min, *p* < 0.01). Wearing the suit led to an increased step width in younger participants (young: 8.7 cm vs young + GERT: 12.1 cm, *p* < 0.01), and did not differ between younger subjects without wearing GERT (*p* = 0.376) and older subjects (*p* = 0.376). Lateral symmetry (see explanation in method section) did not differ between any of the groups (young: -0.2 ± 5.9, young + GERT: 0.6 ± 16.4, older: -0.8 ± 14.7). Wearing the age simulation suit led to a decreased stride length in young subjects (young: 132.6 ± 5.9 cm, young + GERT: 118.1 ± 6.6 cm, *p* < 0.001), but did not match the older subjects (older: 94.5 ± 5.6 cm, *p* = 0.029). Stride time of young participants wearing the suit was different compared to not wearing the suit (young + GERT: 1,316 vs young: 1,172 ms, *p* < 0.001) and different compared to older subjects (young + GERT: 1,316 vs older: 1,152 ms, *p* < 0.01). However, the stride time between the normal gait of younger and older subjects did not differ (young: 1,172 vs older: 1,152 ms, *p* = 0.525).

**Table 2:** Differences between groups in the spatiotemporal gait parameters

Variable	Younger	Younger + GERT	Older
Velocity (km/h)	4.1 ± 0.7^ooo^	3.3 ± 0.5^yyy^	2.9 ± 0.6
Cadence (step/min)	103.5 ± 4.2	92.4 ± 4.1^yyy ooo^	105.2 ± 4.7
Step width (cm)	8.7 ± 2.2	12.1 ± 2.2 ^yyy^	11.1 ± 4.3
Lateral symmetry (mm)	-0.2 ± 5.9	0.6 ± 16.4	-0.8 ± 14.7
Step length left (cm)	66.5 ± 3.7 ^ooo^	59.3 ± 3.8 ^yyy oo^	47.3 ± 3.5
Step length right (cm)	66.1 ± 3.5 ^oo^	58.7 ± 3.7 ^yyy^	47.2 ± 3.2
Stride length (cm)	132.6 ± 5.9 ^ooo^	118.1 ± 6.6 ^yyy o^	94.5 ± 5.6
Step time left (ms)	578.4 ± 89	659.4 ± 56.5 ^yyy oo^	582.8 ± 152.6
Step time right (ms)	585.3 ± 149.4	656.5 ± 98.3 ^yyy oo^	569.2 ± 130.1
Stride time (ms)	1172.7 ± 215.1	1316.4 ± 151.3 ^yyy ooo^	1152.1 ± 219.3

Significance levels: young + GERT vs. young marked with “Y”, *p* < 0.05 (^Y^), *p* < 0.01 (^YY^), *p* < 0.001 (^YYY^); young or young + GERT vs. older marked with “O”: *p* < 0.05 (^O^), *p* < 0.01 (^OO^), *p* < 0.001 (^OOO^))

**Fig. 3:**
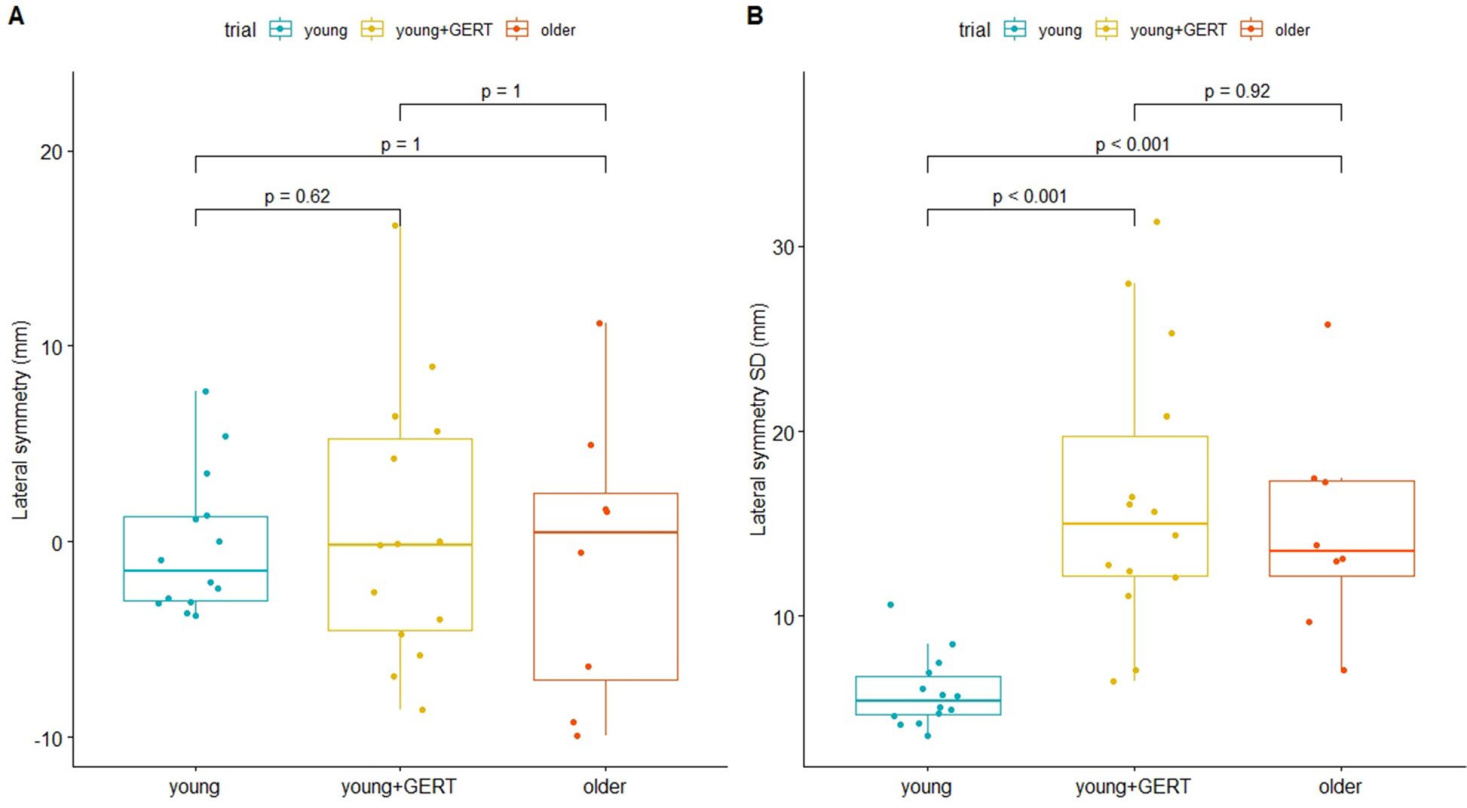
Lateral Symmetry in young subjects with and without age simulation suit compared to older subjects. **A** Differences in lateral symmetry; (**B**) Standard deviation of lateral symmetry between groups. Lateral symmetry did not differ between groups, whereas the standard deviation of lateral symmetry increased by wearing the age simulation suit (young: 5.89 +—1.9 mm, young + GERT: 16.4 +—7.4 mm) and showed no significant difference compared to the older group (old: 14.6 +—5.7 mm)

Some parameters that express gait variability were significantly different between young subjects with and without the age simulation suit (Table 3). Differences were observed in the COV of the step length left (*p* < 0.05), step length right (*p* < 0.05), stride length (*p* < 0.05), step time right (*p* < 0.05) and stride time (*p* < 0.05). No differences were observed for cadence (*p* = 0.66), step width (*p* = 0.17) and stride time left (*p* = 0.08). No differences were observed in any COV parameter between the younger and older and between the younger + GERT and older group. The COV of velocity was not assessed, due to very limited natural variability while walking, because the walking speed was set at the beginning of each trial and did not change during walking. The standard deviation of lateral symmetry, however, differed significantly between younger subjects without and with the age simulation suit (young: 5.89 ± 1.9 mm, younger + GERT: 16.4 ± 7.4 mm, *p* < 0.001) as well as between young subjects without the suit and older subjects (older: 14.6 ± 5.7 mm, *p* < 0.001). However, the standard deviation of lateral symmetry did not differ between younger subjects wearing the suit and older subjects (*p* = 0.525) (Fig. 3).

**Table 3:** Variability of gait parameters (coefficient of variation, COV)

Variable (COV)	Younger	younger + GERT	older
Cadence	4.1 ± 1.1%	4.4 ± 1.1%	4.4 ± 1.5%
Step width	29.9 ± 12.3%	24.8 ± 5.7%	25.3 ± 11.7%
Step length left	5.6 ± 3.5%	6.4 ± 1.9% ^y^	7.6 ± 2.7%
Step length right	5.3 ± 1.9%	6.4 ± 1.6% ^y^	7.3 ± 2.2%
Stride length	4.5 ± 1.4%	5.6 ± 1.7% ^y^	6.2 ± 2.2%
Step time left	14.7 ± 13.9%	8.0 ± 7.3%	25.4 ± 29.1%
Step time right	26.1 ± 21.3%	15.5 ± 16.2% ^y^	22.8 ± 19.2%
Stride time	18.6 ± 10.8%	11.6 ± 8.1% ^y^	18.8 ± 13.3%
Lateral symmetry (regular SD)	5.89 ± 1.9 mm ^OOO^	16.4 ± 7.4 mm ^yyy^	14.6 ± 5.7 mm

Significance levels: young + GERT vs. young marked with “Y”, *p* < 0.05 (^Y^), *p* < 0.01 (^YY^), *p* < 0.001 (^YYY^); young or young + GERT vs. older marked with “O”: *p* < 0.05 (^O^), *p* < 0.01 (^OO^), *p* < 0.001 (^OOO^)

## Discussion

The present pilot study aimed to determine the effect of an age simulation suit on spatiotemporal gait parameters of healthy young adults and compare these parameters with healthy older controls without wearing such a suit. It was hypothesized that wearing an age simulation suit changes the gait characteristics of young people. Furthermore, it was hypothesized that the age simulation suit-induced gait characteristics of young subjects approximate those of the healthy older controls.

Wearing the suit reduced the walking speed (4.1 ± 0.7 to 3.3 ± 0.5 km/h) and the number of steps per minute (from 103 to 92 steps/min) in the younger group. Furthermore, the step length, stride length, step time and stride time were reduced by wearing the suit. However, not every change matched the healthy older controls. Velocity (km/h) was the only marker that differed between older and younger subjects before wearing the suit, but not when younger subjects wore the age simulation suit. The step length for left and right foot and the stride length trended towards the older controls but were still significantly different.

Our study gave similar results to the step length and stride length they seem to decrease with age [3], but both step and stride length were reduced compared to other results [12, 13]. Similar to the parameters mentioned previously, the differences observed for velocity and cadence in younger and older participants are supported by other findings, but absolute values for velocity and cadence in this current study are also lower than those which others had found before [13]. Temporal gait parameters assessed in this study (step time left and right, stride time) did not differ between younger subjects without the suit and older subjects. Our results are in contrast to the results of [3], where age differences in temporal gait parameters were found.

We found differences in the walking speed between healthy young and older individuals; this is in line with published studies [12, 13]. Compared to the study by Chiu and Lusardi, young subjects in our study had a slower walking speed,the walking speed of older individuals was comparable.

We did not find a significant difference in the step width between younger and older subjects, which is in line with previous research for healthy individuals [13, 14]. Only one study [9] assessed gait characteristics while wearing this particular age simulation suit (GERT). Compared to their results, our young subjects walked considerably more slowly (3.1 vs. 4.3 km/h), showed a decreased step length (58.7 vs. 69.7 cm), increased step times (656 vs.550 ms) and comparable step widths (12.1 vs.11.5 cm). While we used a treadmill, [15] assessed walking on a ground-level surface with a length of four meters (GAITRite®).

Regarding markers of gait variability, we found significant differences between COV of step length left and right, stride length, step time left and stride time between young individuals with and without the age simulation suit. No significant difference was observed in the COV of any variable between younger and older subjects or between younger subjects wearing the suit and older subjects. Markers of gait variability seem to be highly variable between individuals and are rarely assessed in younger adults, which makes it difficult to compare these findings to others [13]. The standard deviation of lateral symmetry (a marker specific to this Zebris treadmill), which also serves as a marker of gait variability in this study, showed an increase in younger subjects when wearing the age simulation suit on the same level as the older subjects.

Some of the results might be explained by the methodological framework of the study. Firstly, this study assessed the effect of an age simulation suit on participants walking on an instrumented treadmill. The naivety of subjects towards walking on a treadmill has not been assessed and the relatively long familiarization phase of about five minutes was implemented to reduce the risk of falling or instability regarding walking on the treadmill. It is highly likely that our young subjects are not naïve to treadmills. The walking speed was self-selected and did not change after the initial velocity was achieved and the walking speed of young individuals was similar to the results of other experiments when walking on an instrumented treadmill [16]. However, the same study found subjects walked faster when they had the chance to change the walking speed while walking on the treadmill. This might be a possible explanation of why subjects in this study walked slower than assessed elsewhere. The most important spatiotemporal gait parameters seem to be reliable when assessed on the treadmill used in this current study as stated by Faude et al. [17]. On the contrary, parameters of gait variability inherited less reliability and should be carefully interpreted. In addition the treadmill, it is also important to consider the suit itself. A slower walking speed in younger subjects with the age simulation suit was achieved by increasing the stride time more than in the older group as well as decreasing the stride length to a small extent. Regarding some parameters (e.g., cadence), the suit might have added more mechanical resistance and stiffness than the older participants experienced, whereas other gait characteristics of older individuals were mimicked quite well when wearing the suit. Since we only assessed one specific age simulation suit [18], the results reported are limited to this specific age simulation suit and must not be generalized to different implementations of age simulation suit. It should be noted that wearing the age simulation suit was not reported to be uncomfortable for any of the younger participants regardless of the height or weight of the individual subject. Lastly, differences between groups were assessed with the appropriate statistical tests.

We want to point out that our study design may have influenced our results in several regards: first, the young adults had to perform two consecutive sets of 15 min treadmill walking, in all cases first without and then with the age simulation suit, whereas the older adults only walked once for 15 min. This may have reduced the difference between the groups because of familiarization of the younger adults to treadmill walking. Our method unfortunately does not allow us to re-analyze shorter time fragments, so that we cannot analyze how familiarization (e.g. in the second or third 5 min subsegment) affects outcome. Second, the younger adults have a high level of physical activity (5.9 ± 3.8 yours sport per week). Therefore, they may have shown less difference between normal and age simulation suit than a young adult with lower fitness level. Third, our sample size was smaller than planned (initially planned for 15 participants in each group): we had to stop recruitment due to restrictions during the covid 19 pandemic (study subjects were not allowed within the hospital.) Because of the small sample size, power analyses have not been conducted. Finally, it should be noted that the adaption capabilities to changes in biomechanical parameters can have a large impact on handling the additional influences of the age simulation suit Shadmehr et al. [11] stated that especially young healthy subjects can quickly adjust their locomotor behavior to environmental changes. However, older subjects, who are naturally affected by gait deterioration due to aging processes, do not lose the ability to recalibrate their locomotor system to changes [19]. Moreover, older subjects are used to continuously adapt their gait behavior to possibly highly non-linear changes in movement capabilities [10] showed no significant difference in adaption capabilities between young an older subjects; this is a controversial discussion also in current research. As part of our future work, we will redesign the study protocol in order to investigate the temporal dependencies of adjusting the individual gait behavior to the age simulation suit-induced gait impairments.

In summary, this study reports that wearing this specific age simulation suit during walking on an instrumented treadmill alters most of the spatiotemporal gait parameters of healthy younger individuals, but not every parameter shifts towards gait characteristics of healthy older adults. Regarding gait variability, the standard deviation of the in-house marker of the instrumented treadmill was the single significant difference between younger and older participants. The fact that we only tested on treadmill does not allow us to extrapolate on more complicated, closer to real life gait challenges Testing age simulation suits on normal, uneven or inclining ground would add scientific evidence to the already widespread use of age simulation suits within the education of medical students, nurses, physiotherapists, and others.

## Conclusion

We investigated the effects of an age simulation suit on spatiotemporal gait parameters of young adults and compared them to older, community-dwelling individuals. The measurements were conducted in the movement laboratory of the Clinic for Geriatrics in Aachen, Germany. A treadmill walking trial was performed by each young and older participant; each young participant additionally to normal walking conditions also wearing the age simulation suit GERT. To verify if the suit really simulates aging, we compared standardized gait parameters (measured on a treadmill) to a control group of unaffected older individuals. We could show that the age simulation suit altered some gait parameters significantly towards the characteristics of older individuals’ gait.

Our results support the idea that using an age simulation suit models physiological aging in younger adults: Young subjects were not able to fully compensate for the motion restrictions caused by the age simulation suit, but they were able to maintain stable walking. We interpret this as proof-of-concept to use the age simulation suit for study conception and planning without involving older individuals too early in the process. This could reduce dropout rates, because we would be able to adjust all parameters so that geriatric patients can perform the required steps.

We want to point out that not all investigated parameters changed significantly, so the model is not perfect.

## Data Availability

The data used in this manuscript is available from the corresponding author upon written request.
